# The changing relationship between ENSO and its extratropical response patterns

**DOI:** 10.1038/s41598-019-42922-3

**Published:** 2019-04-24

**Authors:** Nicholas Soulard, Hai Lin, Bin Yu

**Affiliations:** 1 Atmospheric and Oceanic Sciences, McGill University, Montreal, Quebec Canada; 2 Recherche en Prévision Numérique Atmospherique, Environment and Climate Change Canada, Dorval, Quebec Canada; 3 Climate Research Division, Environment and Climate Change Canada, Toronto, Ontario Canada

**Keywords:** Atmospheric dynamics, Climate-change impacts

## Abstract

El Niño-Southern Oscillation (ENSO) can influence Northern Hemisphere seasonal conditions through its interaction with the Pacific-North American pattern (PNA) and the Tropical Northern Hemisphere pattern (TNH). Possibly due to Earth’s changing climate, the variability of ENSO, as well as the zonal location of its sea-surface temperature (SST) anomaly, is changing. Along with this, the strength and location of the jet, in which these atmospheric patterns are embedded, are changing. Using a simple tracking algorithm we create a continuous time series for the zonal location of ENSO’s SST anomaly, and show that the relationship between ENSO and the PNA is linearly sensitive to this location, while its relationship with the TNH is not. ENSO’s relationship with both the TNH and PNA is shown to be strongly influenced by the position of the Pacific jet stream. The ENSO-TNH relationship is found to be linked to phase changes of the Atlantic Multidecadal Oscillation, and the resulting changes in SST and jet speed.

## Introduction

El Niño-Southern Oscillation (ENSO) is a major driver for extratropical Northern Hemisphere interannual variability, especially during the winter months. By inducing convection anomalies in the tropics, it can force atmospheric circulation patterns similar to the Pacific North American pattern (PNA)^1^, and to the Tropical Northern Hemisphere pattern (TNH)^2,3^. The ENSO related sea surface temperature (SST) anomaly in the tropical Pacific sets up Rossby waves that propagate into the mid-latitudes, the structure of which is dependent on the location of ENSO’s SST anomaly, as well as the mean zonal flow^4–7^. Therefore, any change associated with any of the background conditions listed above has the potential to alter the teleconnection between ENSO and the extratropical Northern Hemisphere. Furthermore, these changes in the background state have also been found to be linked to anthropogenic greenhouse gas forcing^8^.

**Figure 1:**
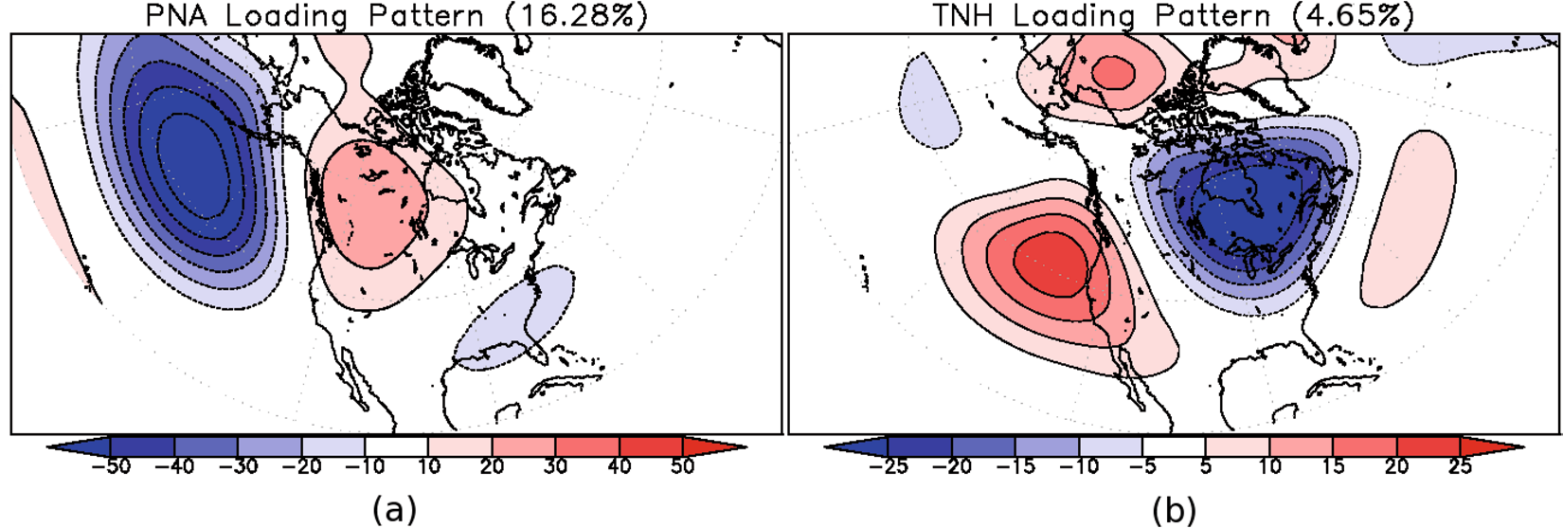
(**a**) The loading pattern associated with the Pacific North American pattern (PNA), and (**b**) the loading pattern associated with the Tropical Northern Hemisphere pattern (TNH). The values in brackets denote the amount interannual variance explained by each pattern.

Even though both the PNA and TNH patterns have been shown to be associated with the tropical SST variability, they can also be generated through internal atmospheric dynamics^9^. In addition, while these two patterns are similar in general structure and appear as a wave train emanating from the tropical Pacific, they are distinct in that their centres of action are nearly in quadrature with one another (Fig. 1). This has sparked some debate as to whether the PNA or the TNH better represents the extratropical response to ENSO. It has been shown that the strength of the TNH is dependent on the strength of El Niño events but not of La Niña events^10–13^, whereas the PNA has been associated with both phases of ENSO^1,4,14^ and internal atmospheric variability^9,15,16^. More recent studies have also shown that the TNH is forced by El Niño events occurring in the eastern tropical Pacific Ocean^17,18^. Likewise, some studies have reported that the PNA is not associated with ENSO at all^19,20^. These works have argued that the extratropical response to positive ENSO events is not the PNA, as many previous studies have stated^1,4,14^ but rather that it is the TNH that best represents El Niño’s response pattern, the PNA being mainly linked to neutral conditions (i.e. internal atmospheric dynamics)^6,14,19,20^.

It has recently been demonstrated that there is a changing relationship between ENSO variability and the magnitude of the Aleutian Low^21,22^, which is associated with both the PNA and TNH. The most recent few decades are marked with an increased correlation between the Aleutian Low and ENSO, which is argued to be related to the increase in the magnitude of ENSO events, and that the mid-century weak correlation was due to a similar decrease in the magnitude. On the other hand, an observed strong correlation between the Aleutian Low and ENSO in the early part of the 20th century was attributed to internal atmospheric dynamics^21^.

This study aims to go a step further and elucidate the changing relationship between ENSO and the two low-frequency patterns that are historically associated with it, the PNA and the TNH. Unlike the aforementioned study that defined the amplitude of the Aleutian Low as the pressure anomaly in the North Pacific, our study takes into account the fact that the Aleutian Low is associated with multiple low-frequency patterns of atmospheric variability and that the Aleutian Low can thus be displaced. In addition, we consider the change in the location of ENSO’s SST anomaly, given that if the location of the forcing changes then so too does the location of the resultant circulation anomaly^6^. In fact, recent studies have suggested that ENSO events can be divided into two categories, east Pacific (EP) events, and central Pacific (CP) events. EP ENSO is the canonical event, with warmest SST anomalies in the eastern equatorial Pacific, while CP events (also known as ENSO Modoki) are characterized by cooler SST in the eastern and western Pacific, with the warmest anomaly in the central Pacific Ocean^23^. EP events have been linked to the TNH while CP events have been associated with the PNA^17,24^. However, the location of the forcing for the PNA is still debated, with some saying that it does not have a longitudinal preference for its forcing^18^. The reasoning behind the PNA and TNH being associated with different types of El Niño events is due to how the resultant Rossby wave generated from these different forcing locations interacts with the mean-flow. From the basic Rossby wave theory the propagation of a Rossby wave depends on the background zonal velocity^4,5^. The North Pacific centre associated with the PNA is located farther west than the one associated with the TNH meaning that the source of the Rossby wave is also located further west^25^. Furthermore, given that the global oceans are warming and that the SST change with global warming is patterned and not uniform, one may expect the relative frequency of EP and CP events to change. Recent studies examining the trend in ENSO’s SST anomaly location agree that it is shifting towards the central Pacific^26–31^, which suggests that CP events should become more and more frequent. This would then imply that the relationship between ENSO and the TNH should decrease in strength, while the ENSO-PNA relationship may strengthen over time. Additionally, a change in the background flow in which these atmospheric eddies are embedded can also result in the response in the geopotential height differing^4,5^.

Should either the ENSO-PNA or ENSO-TNH relationship be favoured over the other, the temperature and precipitation fields associated with that pattern will be favoured as well. Then during years when the PNA, or TNH, is not correlated with ENSO, their impact on the Northern Hemisphere temperature and precipitation may change as well. Previous studies have shown that the type of ENSO event (EP or CP) as well as the duration of the event, all impact the North American temperature and precipitation differently, with persistent EP events yielding increased precipitation over the United States’ west coast^32,33^. This study will show these changing relationships between ENSO and its associated response patterns and put forth a likely mechanism for the changes already observed, be it changes in ENSO’s variability, SST anomaly location, or changes in the background mean flow. Finally we will also comment on the implications for the precipitation and surface air temperature response over North America for the periods of interest.

## Results

### ENSO amplitude

**Figure 2:**
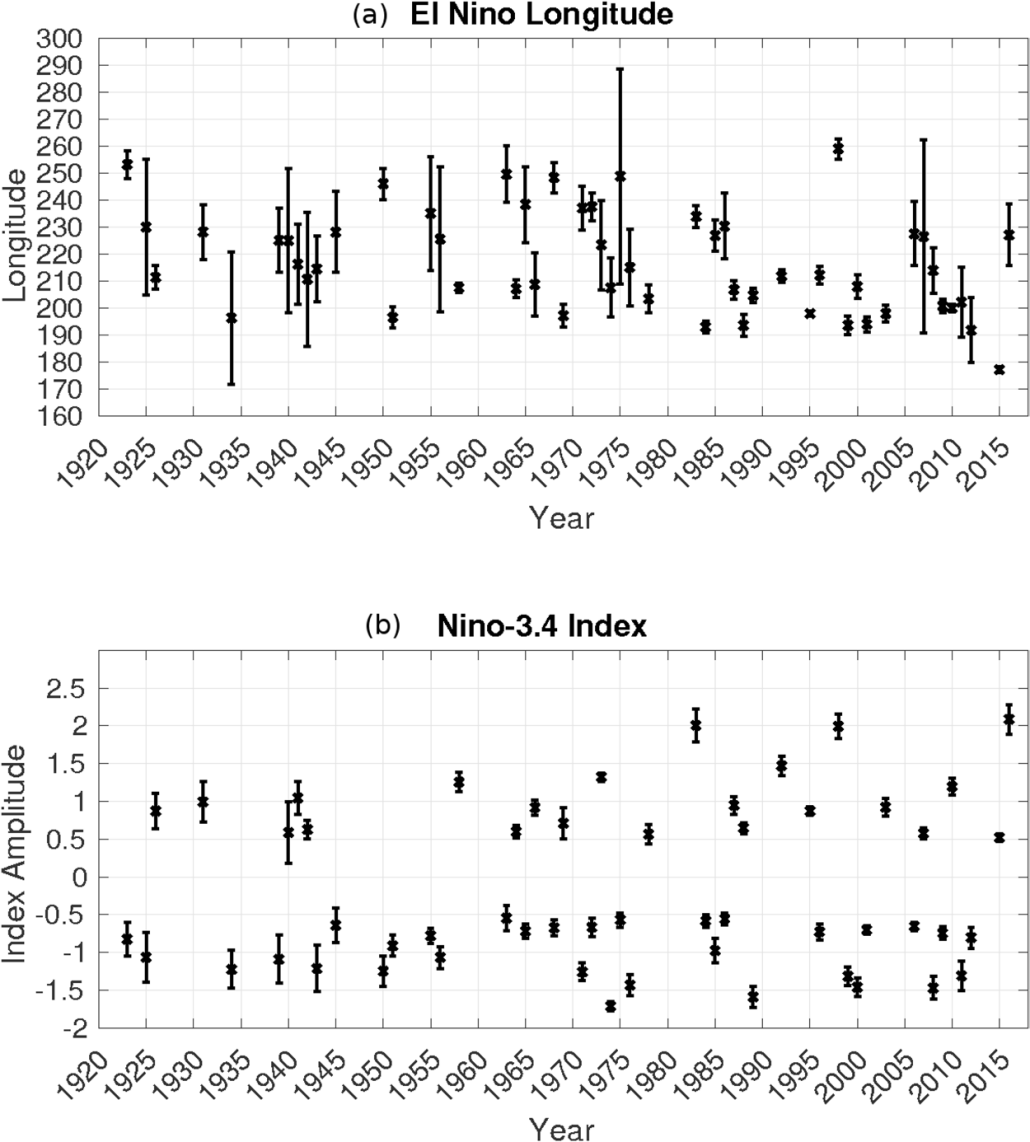
(**a**) The central longitude of the 25° box containing the maximum SST anomaly (see Methods section for details), and (**b**) the Niño 3.4 index. The “x” and bars represent the multi-dataset mean and standard deviation, respectively. Only values surpassing a Niño-3.4 index of half a standard deviation are shown.

Figure 2 presents the mean and inter-dataset standard deviation for the zonal location of the SST anomaly and the amplitude of the ENSO events during the winter months (December through February; see methods). This figure only depicts events that surpass half a standard deviation in amplitude. This is because the tropical SST anomaly is near zero for weak events, and since our tracking algorithm locates the largest SST anomaly that has the same sign as the Niño-3.4 index, the results can be spurious for weak events since the largest anomaly may not be related to ENSO variability, and hence not necessarily reflect the location of ENSO related anomalies. The spread for both the amplitude of an event and its location tends to decrease from past to present (Fig. 2). This decrease in spread, or increase in dataset agreement, is likely due to the addition of more abundant and accurate measurements. It is also noted that the events with a larger inter-dataset spread are generally relatively weak events.

**Figure 3:**
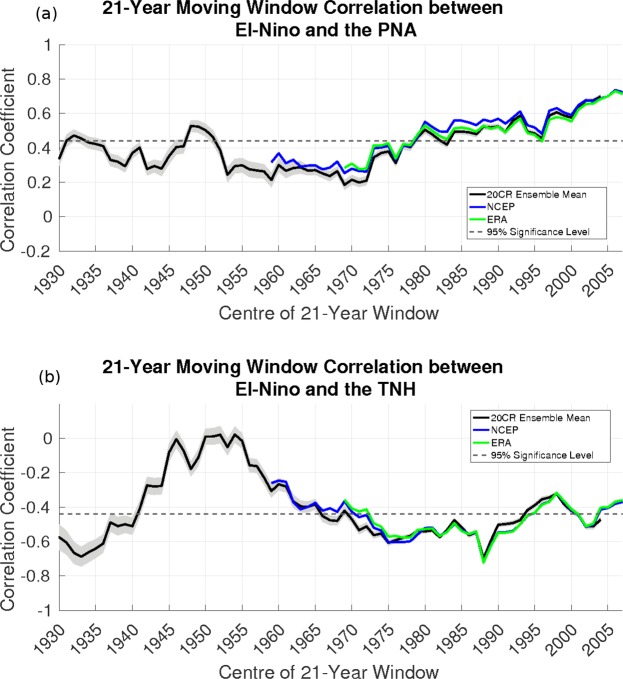
The 21-year moving window correlation between the Niño-3.4 index and the (**a**) PNA index, and (**b**) TNH index. The Values above (below) the dashed line surpass the 95% significance level for the correlation associated with the PNA (TNH). The green line is the combined ERA-Interim/ERA-40 dataset, the blue line is the NCEP/NCAR reanalysis, and the black line with grey shading is the mean and ensemble standard deviation of the 20^th^ century reanalysis.

In general, the amplitude, and hence variance, of ENSO events tends to increase with time, with the three largest ENSO events on record occurring in the last few decades (Fig. 2b). Examining the correlations between the Niño-3.4 index and the PNA and TNH indices, we find that the multi-dataset average correlation for the TNH (PNA) is −0.51 (0.61) when considering only events surpassing half of a standard deviation, consistent with previous studies showing the link between ENSO and the extra-tropics^1,4,10–14^. The larger proportion of strong ENSO events in the late 20th and early 21st centuries explains why we see a general increase in the strength of these relationships (Fig. 3).

### ENSO SST location

Previous studies indicate that the PNA and TNH have each been linked to different types of ENSO events; the PNA to CP events^17,24^, and the TNH to EP events^10,13,17,18^. CP events have become more frequent in recent decades^26–29^, as also seen in Fig. 2a, hence we expect that the relationship between ENSO and the PNA will strengthen over time, while its relationship with the TNH will weaken. Figure 3 depicts the 21-year moving window correlation between ENSO and the time series of each of the two atmospheric patterns. All three datasets containing both atmospheric and SST data (see Table 1) show the same trends and very similar magnitudes for the correlation between the PNA and TNH indices and the Niño-3.4 index. Clearly evident is the increase in the correlation between the PNA and ENSO time series from the early 1970 s to present. In the early part of the 20^th^ century, the correlation between them was fairly variable and insignificant until the late 1950 s. Since the SST anomaly associated with ENSO has begun to move westward into the central Pacific Ocean, the correlation has been increasing, reaching a maximum of about 0.7 in recent years. The coincidence of these two trends lends support to previous studies linking the PNA to CP ENSO variability. In order to ascertain with any certainty whether the strength of the ENSO-PNA relationship is influenced by the longitude of the SST anomaly, we apply a 21-year moving window mean to the ENSO longitude, excluding weak years, and then compute the correlation between this time series and that of the ENSO-PNA relationship. This yields a correlation of −0.73 using the 20^th^ century reanalysis data, which suggests that when the SST anomaly is located more towards the central Pacific (smaller longitude anomaly) then the strength of the teleconnection between ENSO and the PNA increases, consistent with previous studies^17,24^. These values are similar in the other datasets: −0.94 in NCEP/NCAR, and −0.80 in the combined ERA-Interim/ERA-40.

**Table 1:** Listed are the various datasets employed in this study, along with the general type of data used (atmospheric fields or SST), the time span considered, and the reference associated with each dataset.

Variable	Data source	Time Span	Reference
Atmospheric fields and SST	20th Century Reanalysis	1920–2014	^52^
	NCEP/NCAR Reanalysis 1	1949–2017	^53^
	ERA-Interim and ERA-40 combined	1959–2017	^54, 55^
SST only	Extended Reconstructed Sea-Surface Temperature	1920–2017	^56, 57^
	Hadley Centre Ice and Sea-Surface Temperature	1920–2016	^58^
	Hadley Centre Sea-Surface Temperature version 3	1920–2017	^59, 60^
	Optimum Interpolation Sea-Surface Temperature	1981–2017	^61^

By contrast, the correlation between ENSO and the TNH (Fig. 3b) did not show a strong weakening trend in recent decades, as would be expected from the movement of the SST anomaly. In fact, we see a comparable strengthening of their relationship from the late 1950 s until the early 1980 s, a slight weakening from the late 1980 s until the late 1990 s, and a marginal rebound thereafter (Fig. 3b). This result is counter intuitive given that previous studies have found that the TNH is forced by strong EP events. When examining the correlation between the strength of the ENSO-TNH relationship and the 21-year mean SST longitude for the 20^th^ century reanalysis ensemble mean, we find that the correlation is 0.39, which does not pass the 99% significance threshold, and the sign is inverted from what would be expected. The positive correlation implies that for more EP events we would likely see more positive correlation between ENSO and the TNH, contrary to all previous studies^10,13,17,18^. Furthermore, examining this same relationship in other datasets we see a near zero correlation in NCEP/NCAR, and a weakly negative correlation in the combined ERA-Interim/ERA-40 datasets. These suggest that while the TNH can be forced by strong EP events, the relationship between ENSO’s SST anomaly location and the ENSO-TNH relationship is not a linear one.

However, we can see that the SST anomalies associated with each the PNA and TNH overlap substantially (Fig. S1). This coupled with correlation between the ENSO SST longitude and ENSO-PNA correlation indicate that the PNA is associated with a more expansive SST anomaly, which extends further west, than that associated with the TNH. ENSO events centred in the eastern tropical Pacific (Niño-3 region of 210E–270E) produce a structure that resembles the TNH (not shown). On the other hand, events that occur in the central-west tropical Pacific (Niño-4 region of 160E–210E) produce a structure that resembles neither the TNH nor the PNA (not shown). Using this kind of composite based on the longitude of ENSO’s SST anomaly, we were not able to determine whether either the TNH or the PNA is more likely to be associated with EP or CP events. This can be accomplished by plotting the ENSO-PNA/TNH correlation time series against the 21-year running mean ENSO longitude. In decades when ENSO’s SST anomaly stays (on average) west of 210E, ENSO tends to force the PNA pattern (Fig. S2). For the TNH, as mentioned above, the scatter plot is much less linear; however, there is still a range of longitudes that favour the ENSO-TNH correlation. There is a substantial increase in the absolute value of the correlation when ENSO’s SST anomaly stays in the vicinity of 210E–222E. From Figs 3 and S2 we also note that there are a range of SST anomaly locations for which both the PNA and TNH are statistically significantly correlated with ENSO variability. During the 1980 s to 1990 s, for example, both the PNA and TNH were statistically significantly correlated with ENSO. Around this time period CP ENSO events began to become more frequent (Fig. 2). These changes in ENSO’s SST location may be due to multi-decadal variability (see section with same title) or due to internal dynamics. However, the transition from more EP to more CP events likely led to a period of transition from the ENSO-TNH relationship being dominant to the ENSO-PNA relationship being the dominant one. Given that previous studies have documented that EP ENSO events force the TNH and CP ENSO events force the PNA, it seems likely that an ENSO event where the SST anomaly lies between EP and CP, may force some sort of pattern which spatially projects onto both the PNA and TNH. This can be confirmed using a simple GCM linearised about the observed boreal winter basic state, which is disturbed by an imposed diabatic heating anomaly in the Tropics^34^. When the imposed forcing is centred closer to the dateline (200E forcing) the response in the extra-tropical Northern Hemisphere resembles a PNA-like pattern with an Aleutian Low centred near the dateline, and anomalies of alternating signs arching across North America. As the diabatic heating anomaly is imposed further and further east (towards 225E) the response becomes more TNH-like with the Aleutian Low pressed against the North American west coast. In the intermediate heating positions (from 210E to 215E) the Aleutian Low begins to move eastward and thus its position overlaps with both the PNA and TNH response patterns. Furthermore, the North American high pressure centre is also shifted eastward (Fig. S3).

### Zonal jet variability

**Figure 4:**
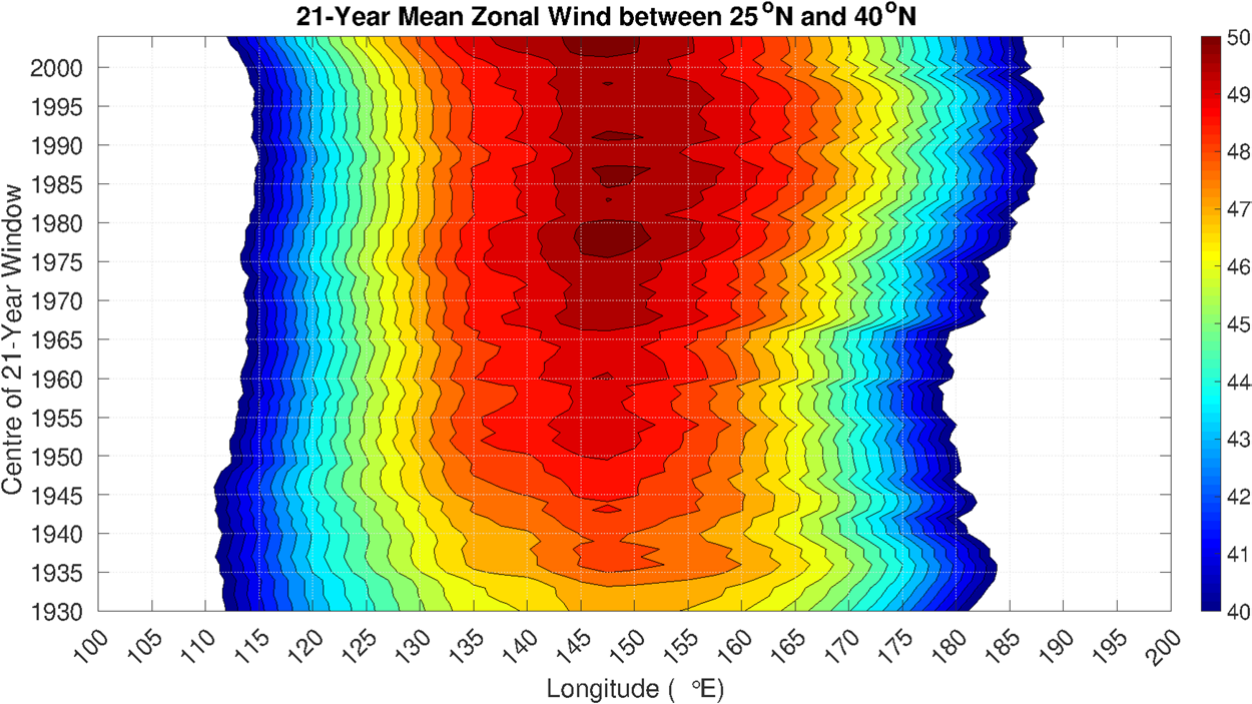
21-year moving window mean zonal wind speed averaged between 25°N and 45°N, and 120°E and 240°E for the 20^th^ century reanalysis. The contour interval is 0.5 m/s with an outer contour of 40 m/s.

Another factor that can impact the relationship between ENSO and the PNA and TNH patterns is changes in the background flow. The relationship between the background wind and the correlation between ENSO and the TNH and PNA is further explored by examining the mean zonal wind in these same 21-year windows. In general, when growing disturbances are zonally elongated in the presence of mean flow diffluence, the mean flow imparts energy to the disturbances^15^. This is usually the case in the vicinity of the Aleutian Low. However, the TNH is associated with an Aleutian Low that is displaced to the east, towards the west coast of North America. In this location, the Aleutian Low is slightly removed from the mean flow’s region of maximum diffluence in the jet exit region. To this end, we compute the average wind speed in the midlatitude Pacific jet region, between 25 and 45 degrees North, in moving 21-year windows (Fig. 4). Following a given isopleth (say 40 m/s, the outer contour), we note that the eastern edge of the jet remained relatively stationary between the early 1940 s and 1970, after which it began to pulse eastward until the 1990 s. The jet then retracted back westward between the late 1990 s and present. This pattern of jet position matches the variations in the relationship between ENSO and the TNH with a correlation of −0.55 over the entire record of the 20^th^ century reanalysis. This suggests that it is the eastward pulse of the Pacific jet that acts to impart relatively more energy from the mean flow to the TNH variability. Thus for a given SST forcing, in years when the jet extends anomalously far to the east we can expect a stronger TNH pattern. This can be shown by plotting energy exchange between the mean zonal flow and the eddies associated with the TNH (see Methods; Fig. S4). Similarly, since the Aleutian Low associated with the PNA is located farther west, it too is associated with the movement of the jet (correlation of 0.71). Moreover because of the collocation of the Aleutian Low associated with the PNA and the jet maximum, the Aleutian Low is in the proximity of the jet exit region, where diffluence is the strongest. Therefore the strength of the relationship between ENSO and the PNA is correlated with the mean zonal wind speed (corr = 0.56) as shown by the central magnitude in Fig. 4, indicating increasing diffluence strengthens the teleconnection.

### Multi-decadal variability

**Figure 5:**
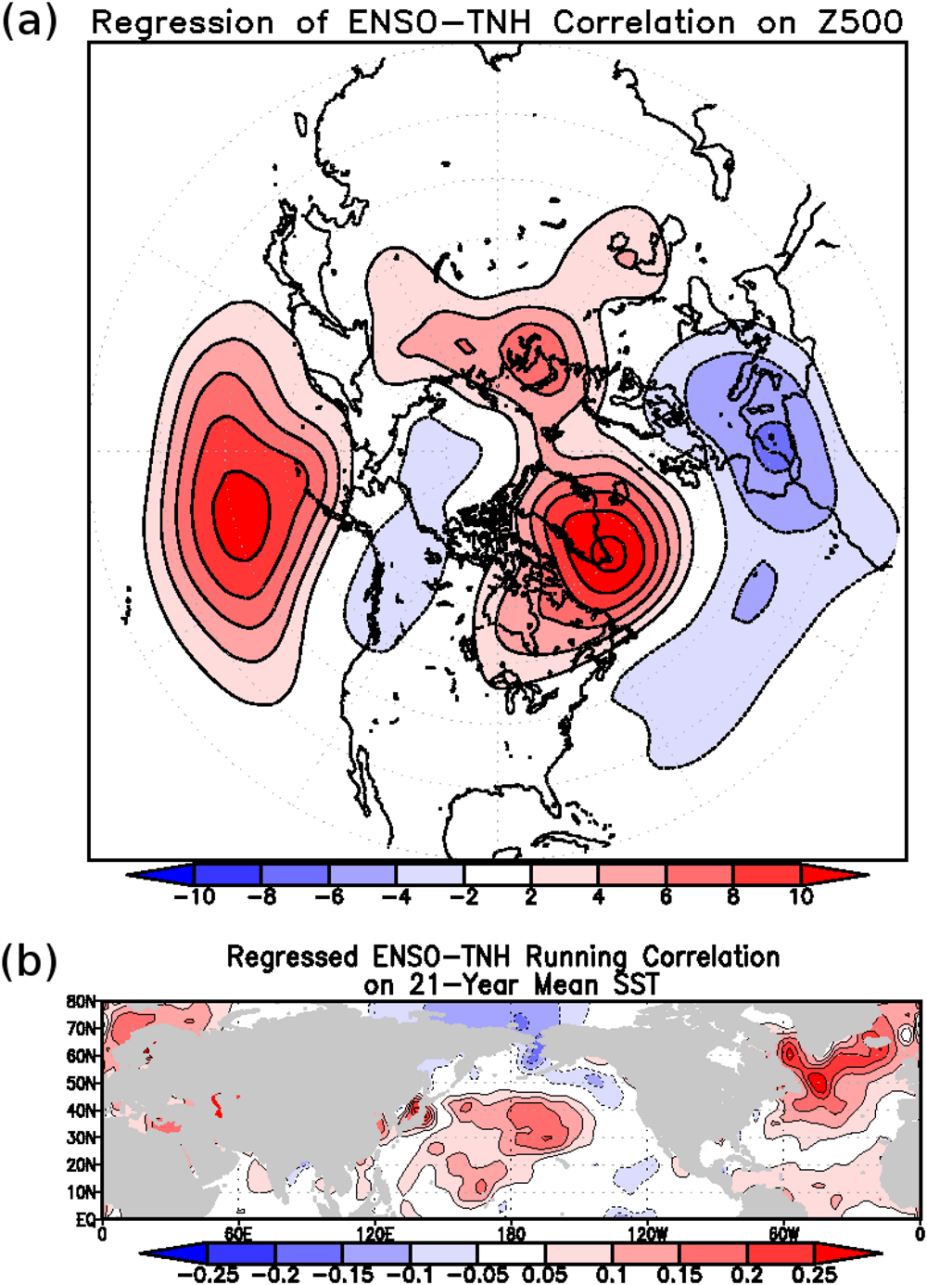
(**a**) The 500 hPa geopotential height pattern, in units of metres, associated with the ENSO-TNH running correlation time series. (**b**) Regression of the ENSO-TNH correlation time series against Hadley SST Version 3. The units are in degrees Celsius. Only values surpassing the 95% confidence level are shaded.

The changes in the teleconnection between ENSO and its associated response patterns can potentially be modulated by longer time scale variability, such as the Pacific Decadal Oscillation (PDO)^35^ and/or the Atlantic Multidecadal Oscillation^36^. To examine this further we regress the ENSO-PNA/TNH running correlation time series against global SST. Any pattern that emerges from this is likely on a longer time-scale than either ENSO, or its response patterns, since there is a low-pass filter inherent in the 21-year running correlation. Therefore any anomaly pattern that emerges likely exerts some modulating influence on the relationship between ENSO and its atmospheric response patterns. This regression for the ENSO-PNA time series yields no meaningful results (not shown). The global SST anomaly field is both very weak and insignificant. By contrast the ENSO-TNH relationship is associated with strong positive SST anomalies surrounding the south of Greenland (Fig. 5b); a pattern reminiscent of the AMO. Recently, there have been studies showing that the multi-decadal variability in the Atlantic can exert some influence in the Pacific basin^37,38^. When looking at the correlation between the 21-year moving mean AMO index (derived from Hadley SST version 3 data as in^39^) and the ENSO-PNA/TNH time series, we find a clear correlation between the AMO and the ENSO-TNH relationship, but not the ENSO-PNA one. The correlation between the 21-year mean AMO and the ENSO-TNH correlation ranges from 0.47 (20CR) to 0.76 (NCEP) depending on the dataset used, these values are all quite high and statistically significant at the 99% level. A previous study has posited a possible connection between the AMO and Pacific basin, suggesting that during positive AMO phases the storm track in the North Pacific is weakened, and results in a weaker poleward shifted jet^40,41^. This weakening and shifting of the jet is due to the atmospheric circulation pattern induced by the AMO, which has a strong positive centre over Pacific basin (see Fig. 5a; pattern consistent with previous study^41^). The modification of the background wind field leads to an unfavourable environment for the TNH (see Fig. S4) and thus a weakening of the ENSO-TNH relationship. During the negative phase of the AMO, the opposite is true, and the ENSO-TNH correlation strengthens.

## Summary and Conclusion

These changes in the relationship between ENSO and its extra-tropical response patterns also lead to changes in ENSO’s impact on temperature and precipitation over North America (Fig. S5). During positive PNA winters when it is only weakly associated with ENSO, its impact on North America is to warm Canada and the western United States, cool the south eastern United States, while weakly diminishing the amount of precipitation the northwestern and central east United States receive. During years when the ENSO-PNA correlation is high, positive PNA events lead to more ubiquitous warming over Canada and the United States, with weak cooling in the southeastern United States. The states around the USA/Mexico border see increased precipitation, while the northwest still sees a diminished amount of precipitation relative to the mean. When the ENSO-TNH relationship is stronger, negative TNH events result warming mainly over Canada and the northwestern United States. Correspondingly it is associated with increased precipitation centred over California and Florida. During years when the ENSO-TNH relationship is weak, the TNH does not impact precipitation over North America, but does warm eastern Canada. These results are similar to those in previous studies, which show that for strong, sustained EP ENSO events, the west coast should see relatively more rainfall than during CP events due to the location of the circulation anomalies associated with the TNH and PNA, respectively^32,33^.

It is shown that the relationship between ENSO and each the PNA and TNH strengthens with increasing SST variability, as would be expected, since a stronger forcing should linearly yield a stronger response. Examining the location of the SST anomaly associated with ENSO shows that the location of ENSO’s SST anomaly is trending more towards the central Pacific, resulting in an increased frequency of CP events in recent decades. Using multiple datasets, we show that during decades with more CP events the teleconnection between ENSO and the PNA is strengthened. The SST anomaly’s location is found to be statistically significantly correlated with the strength of the ENSO-PNA relationship, suggesting that the westward trend in ENSO SST anomalies is partly responsible for the strengthening teleconnection. By contrast, this trend does not appear to linearly affect the ENSO-TNH relationship. The ENSO-TNH relationship is favoured for SST anomalies centred around 215E–220E. Therefore, even though the correlation between the ENSO SST location and the ENSO-TNH relationship isn’t statistically significant, the scatter plot reveals that a relationship does exist (Fig. S2).

The Pacific jet stream is found to play an important role in influencing ENSO’s extra-tropical responses. When the jet extends further east, it is able to impart more energy to the Aleutian Low associated with the TNH. This correlation indicates that during decades when the jet extends eastward, the ENSO-TNH relationship is strengthened. The ENSO-PNA relationship seems to be related to the zonal wind as well, however, the strength of this relationship is due to both the wind speed and location. The reason being that the Aleutian Low associated with the PNA is located far enough west that it is in the vicinity of the jet max, and so variations in the jet’s eastward extension as well as its speed influence the amount of energy the mean flow can impart. In contrast, the Aleutian Low associated with the TNH is located off the west coast of North America, just at the edge of the jet, so only variations in its position can change in the amount of energy imparted, and a change in the strength of the ENSO-TNH relationship.

It appears as though one of the factors modulating the jet is the AMO. Previous studies have indicated that during the negative phase of the AMO, EP ENSO events are favoured, and the jet is strengthened and displaced equatorward with respect to the mean. These changes to the background state create a favourable environment for the relationships between ENSO and the TNH. However the ENSO-PNA relationship does not appear to be modulated by the AMO, but solely by the trend towards more and more CP events.

## Future Considerations

All of these relationships are complicated by the fact that while the primary driver for PNA and TNH variability is ENSO, other forcing mechanisms have the potential to affect these relationships. Typically, the extratropical SST is found to be associated with, and lag behind, atmospheric anomalies^42,43^. For example the cold anomaly in the North Pacific associated with both the PNA and TNH, has been found to be a response to the circulation of the Aleutian Low^44^. However, this does not mean that the interaction is one-way. For example, during an ENSO event, the Walker circulation shifts eastward and results in subsidence over the Indian Ocean. This subsidence leads an increased SST anomaly in the region^45^. A warm SST anomaly in this region produces a PNA-like response of the opposite sign as the ENSO anomaly, and thus counteracts the ENSO forcing^46^.

While we make use of a simple linear GCM to support our results, a more comprehensive modelling study, utilizing an ensemble of fully coupled climate models, would allow for a more detailed examination of the roles of both internal and external variability, along with isolating the role of Tropical Pacific forcing in comparison to extratropical modulating factors. For example, one important question to address is to what extent is the movement of ENSO events from mostly EP to mostly CP determined by external variability, like the AMO, and internal variability.

## Methods

We make use of several long time-span reanalysis and observational SST datasets, as well as several datasets with additional geopotential height and zonal wind data (Table 1). All data presented herein is for the winter mean (Dec-Feb), and anomalies are with respect to the 1981–2010 base period. All statistics computed herein are computed for each dataset (and ensemble member in the case of the 20^th^ century reanalysis) individually. Given that these datasets each employ differing data assimilation techniques, and have differing degrees to which they depend on model output, consistency between the various datasets is a sign that the signals seen herein are not simply artefacts of the observational system used.

While there are some studies that make use of a continuous location index^47^, the majority of studies that examine the different types of ENSO characterize EP events and CP events using either area-averaged anomalies or loading patterns^17,26,29,48,49^. When employing these indices we obtain a bimodal distribution with very little information on the actual location of the SST anomaly; either it is an EP event or a CP event. To better represent the variability of the location of ENSO’s SST anomaly, we construct a continuous time series that depicts the zonal location of the SST anomaly in units of degrees East. We implement a simple tracking algorithm to estimate the location of the SST anomaly. This index is constructed as follows: for every year we compute the average SST anomaly from 5°N to 5°S, and over a 25° longitudinal span. A 25° longitude span is chosen so that the box is wide enough to smooth out any single grid point with an abnormally high anomaly and average over a sufficient area to capture the ENSO anomaly centre, and yet small enough to roughly capture the largest portion of the ENSO SST anomaly. We move this 25° box over by one grid point (2.5°) and take the average again, and so on, until we have sampled the entire equatorial Pacific. We then extract the box with the largest SST anomaly of the same sign as the concurrent Niño-3.4 index and note the central longitude of this 25° box. This index allows us to verify whether the various datasets are consistent in their representation of the location of ENSO related SST anomalies. Since there are very few constraints imposed on identifying the ENSO anomaly centre, we can identify events where the anomaly might be strong but geographically small. Additionally, it allows us to discover whether the location of the SST anomaly is moving over time, and compute statistics using this time series (Fig. 2a). Amplitude can also be extracted during this process, as the amplitude of the SST anomaly in said box. However while its value differs slightly from the Niño-3.4 index (Niño-3.4 tends to have a smaller amplitude, since the box is larger), the correlation between the two is quite high for all the datasets (>0.9). We therefore elect to employ the more commonly used Niño-3.4 index when discussing the amplitude of ENSO (Fig. 2b), while noting that all our results are consistent using either index.

To then shed light on the changing relationships between ENSO and its associated low-frequency patterns of variability, we compute a 21-year moving window correlation between the Niño-3.4 index and the PNA and TNH indices for each dataset. The TNH and PNA indices are derived from a rotated empirical orthogonal function (REOF) analysis of 500 hPa geopotential height anomalies^50^. We first extract the two REOF patterns from the multi-dataset mean for the overlapping years of 1959–2014. This is done so that the resultant patterns, while consistent with those typically presented, also contain a contribution from each dataset, and provides information of which datasets are more or less variable than the mean. The PNA appears as the second REOF pattern, while the TNH as the sixth, explaining 16.28% and 4.65% of the interannual variability, respectively (Fig. 1). The 500 hPa geopotential height anomalies for each year in each dataset are then projected onto these two patterns to obtain the time series for both the PNA and TNH for all three atmospheric datasets. Finally, from the atmospheric datasets, we also examine the 250 hPa zonal wind field in 21-year moving windows to represent the background flow that these patterns are embedded within.

To examine the cause and effect relationship between the mean-flow and eddies, we can look at the exchange of energy between the two. We compute the change in kinetic energy of the eddies as the product of the horizontal component of the extended Eliassem-Palm flux vector with the gradient of the zonal background wind^15,51^.

It is important to note that several of the datasets used in this study extend roughly a century into the past, before a reliable, comprehensive, observational network was established. Results based upon values from this far in the past should be taken with a grain of salt. For the purposes of this study, we make use of several of these datasets, but complement them with more recent ones. Therefore agreement between datasets during the more recent past lends credence to values from farther in the past. It is also important to note, that while we generally present results for multi-dataset averages, the individual datasets each yield similar and consistent results.

To complement the present observational study, we make use of a simple linear GCM, based on the dry primitive equations^34^. The model is balanced by constant winter forcing, and we impose a diabatic heating anomaly along the equator. This heating has a vertically integrated amplitude of 1 Kelvin/day, and peaks at 350 hPa, mimicking tropical convection. This forcing is imposed at various longitudes along the equator to simulate the extratropical response associated with an ENSO-type anomaly at these same locations.

## Supplementary information

Supplementary Material

## Data Availability

The code associated with this paper is available on request from N.S.
