# Supplementary material for: The changing relationship between ENSO and its extratropical response patterns
Source: Sci Rep. 2019 Apr 24;9:6507. doi: 10.1038/s41598-019-42922-3 (PMC6482142; doi:10.1038/s41598-019-42922-3)
Supplement: Supplementary file 1 — Supplementary Material [file 41598_2019_42922_MOESM1_ESM.doc]

**The changing relationship between ENSO and its extratropical response patterns**

Nicholas Soulard^1, *^, Hai Lin^2^, Bin Yu^3^

1. Atmospheric and Oceanic Sciences, McGill University, Montreal, Quebec, Canada

2. Recherche en Prévision Numérique Atmospherique, Environment and Climate Change Canada, Dorval, Quebec, Canada

3. Climate Research Division, Environment and Climate Change Canada, Toronto, Ontario, Canada

* Corresponding Author: Nicholas Soulard

**Email:** [**nick.soulard@mail.mcgill.ca**](mailto:nicholas.soulard@mail.mcgill.ca)

**Supplementary Material**

**Figure List:**

Figure S1. Composite during the periods of (bottom) maximum and (top) minimum correlation (given from Fig. 3 in the manuscript) for the positive (left) PNA events, and (right) negative TNH events during these periods. Units are degrees Celsius.

Figure S2. Scatter plots of 21-year mean ENSO SST longitude against (top) ENSO-PNA running correlation, and (bottom) ENSO-TNH running correlation. Units in metres.

Figure S3. 500 hPa response in a simple linear GCM to an imposed diabatic heating anomaly centred on the equator and the longitude list in each panel’s title.

Figure S4. Energy exchange (shading) between the mean zonal flow and the TNH eddies at 500 hPa (contours). Units of energy in Joules/day.

Figure S5. Similar to Figure S1, but for (top four panels) precipitation anomalies, and (bottom four panels) 2m air temperature anomalies. Units are in mm/day and degrees Celsius, respectively.

Figure S1. Composite difference during the periods of (bottom) maximum and (top) minimum correlation (given from Fig. 3 in the manuscript) for the positive (left) PNA events, and (right) negative TNH events during these periods. Units are degrees Celsius.

Figure S2. Scatter plots of 21-year mean ENSO SST longitude against (top) ENSO-PNA running correlation, and (bottom) ENSO-TNH running correlation.

Figure S3. 500 hPa response in a simple linear GCM to an imposed diabatic heating anomaly centred on the equator and the longitude list in each panel’s title. Units in metres.

Figure S4. Energy exchange (shading) between the mean zonal flow and the TNH eddies at 500 hPa (contours). Units of energy in Joules/day.

Figure S5. Similar to Figure S1, but for (top four panels) precipitation anomalies, and (bottom four panels) 2m air temperature anomalies. Units are in mm/day and degrees Celsius, respectively.
